# Internet searching produces misleading findings regarding violent deaths in crisis settings: short report

**DOI:** 10.1186/s13031-019-0187-z

**Published:** 2019-02-15

**Authors:** Mary Grace Flaherty, Leslie F. Roberts

**Affiliations:** 1 School of Information and Library Science, University of North Carolina, Chapel Hill, NC 27599-3360 USA; 2 Population and Family Health, Mailman School of Public Health, Columbia University, New York, NY 10023 USA

**Keywords:** Violent death rate, Internet searching, Humanitarian injustice

## Abstract

Donor and agency priorities are influenced by a variety of political, social, and media-related forces that can have a profound impact on response and resource provision. We have attempted to assess how well internet searches articulate the span of violent death rates for five current “crisis” settings. In three graduate classes (2 public health, 1 information science) at US universities, during a four month period in 2017–2018, we asked approximately 60 graduate students to conduct an internet search to determine which of five countries had the highest and lowest “violence specific mortality rate”: Venezuela, Syria, Yemen, Central African Republic (CAR), or Mali. Students were divided into groups of three, and within each group explored this question by three approaches. Many graduate students in all groups could not determine the relative rates, especially which country had the lowest violence specific mortality rate. Of the 34 searches that identified a highest violent death rate country, 27.5 (81%) concluded it was Venezuela, followed by Syria (4.5; 13%), Mali (1; 3%) and CAR (1; 3%). Of the 26 searches that identified a least violent death rate 21.5 (83%) reported either CAR or Mali, followed by Yemen (2.5; 10%) and Syria (2; 8%). Aside from lack of data on CAR and Mali, students were perplexed about whether to include suicides or executions in the measure. This resulted in almost half of all inquiries unable to estimate a highest and lowest rate among these five countries. Where conclusions were drawn, it is likely the internet drew students to the opposite conclusion from reality. There are several reasons for this discordance, such as differing categories of violent deaths as defined by the World Health Organization, and search engine algorithms. It is probable, however, that larger issues of connectivity of individual societies with each other and the outside world are playing a profound role in the deceptive results found in this exercise. This insight emphasizes the internet’s under-reporting in the world’s most poor and remote locations, and highlights the importance of primary data collection and reporting in such settings.

## Background

In the world’s worst crises, it is now common that violence is both the driving force and primary cause of death [1]. In 2015, a record $28 billion (USD) was spent on humanitarian relief; yet United Nations Coordinated Appeals were only 55% funded [2]. This suggests the relief community is always operating in triage mode, attempting to operate where conditions are most dire or where aid can provide the most benefit. For many organizations, the primary indicator of a humanitarian crisis is the crude death rate [3]. From an information standpoint, death tolls and estimates of violent death rates can be inaccurate and controversial, often with sources reporting a ten-fold range in violent death rates [4–6]. This can have a profound impact on response, support, and resource provision.

Incorrect information about rates of mortality and rates of violence can arise from many mechanisms. In the case of major international powers, leaders can just deny scientific findings [7, 8]. Political leaders can also cite inaccurate high death or low death tolls from less than rigorous sources to influence or confuse public perception [9, 10]. Sometimes certain deaths are more visible. For example, in recent years, deaths of migrants crossing the Mediterranean from Northern Africa have received a lot of attention even though it is likely far more migrants die en route crossing the Sahel [11]. But more commonly, people who are not well connected to the international community, who may be illiterate, and/or in rural and undeveloped settings, can be killed in large numbers with little international media attention or social record. This happened most recently when approximately 9000 Rohingya in Myanmar were killed; this was not fully appreciated until Médecins Sans Frontières conducted a survey among this population in exile months later [12].

We have attempted to assess how well internet searches articulate the relative rates of violent death rates for five current “crisis” settings.

## Methods

We chose 5 conflict settings: Venezuela, Syria, Yemen, CAR and Mail for assessing the ability of the internet to reflect levels of violence. All of these conflicts have existed for multiple years. Venezuela and Syria were selected as they are relatively wealthy and socially networked societies and receive a considerably high profile in news outlets. CAR and Mali were chosen as violent settings with the lowest and 3rd lowest rankings in the 2015 Human Development Index [13], and where social connectivity to the rest of the world seems low. Yemen was chosen as a high profile conflict that in terms of wealth and social networks appears to be somewhere in-between. No preliminary internet searching was used in the selection of these countries.

During a four month period spanning October 2017–February 2018, we asked graduate students to conduct an internet search to determine which of the five countries had the highest “violence specific mortality rate.” This exercise was performed in three different classes (2 public health, 1 information science), at three separate universities in the United States. These approximately 60 graduate students were asked to break into groups of three students and within each group to explore the question by three mechanisms:

- One search was to be conducted with a general search engine such as Bing or Google.
- One search was to be conducted with a “constrained criteria search mechanism such as Medline or Google Scholar.”
- One search should address this question without starting a specific search, but instead by going to the internet source they deemed most credible for the query (e.g. the World Health Organization, the US Central Intelligence Agency Factbook, or Ministries of Health for specific countries).

Groups were given approximately 20 min to search the internet and assess the relative rates of violent deaths. If a search concluded one of two countries had the highest or lowest measures, each was given 0.5 “counts” for that finding. When questions arose, for example about which of the World Health Organization’s (WHO) 5 categories of violent deaths to include, the facilitators avoided making any clarifications, and left students to make their own judgments.

## Results

Many graduate students in all three groups could not determine the relative rates, especially which country had the lowest violence specific mortality rate. Of the 34 searches that identified a highest violent death rate country, 27.5 (81%) concluded it was Venezuela. This was followed by Syria (4.5; 13%), Mali (1; 3%) and CAR (1; 3%). Of the 26 searches that identified a least violent death rate 21.5 (83%) said either CAR or Mali (often unable to distinguish because WHO presents no mortality data for either), followed by Yemen (2.5; 10%) and Syria (2; 8%). These findings are demonstrated in Table 1.

**Table 1:** Reported Results of Internet Searching for Violence Specific Mortality

Country	# (%) estimated as highest	# (%) estimated as lowest
Venezuela	27.5 (81%)	
Syria	4.5 (13%)	2 (8%)
Yemen		2.5 (10%)
CAR	1 (3%)	21.5 (83%)
Mali	1 (3%)	

Aside from lack of data on CAR and Mali, students were in some cases perplexed about whether to include suicides or executions in the measure. This contributed to almost half of all inquiries unable to estimate a highest and lowest rate from among these five countries.

## Discussion

While not completely certain, it is likely that the internet drew students to roughly the opposite conclusion from reality. Of the 5 countries, Venezuela probably has the lowest violence specific death rate of about 50 per 100,000 population per year [14]. This compares with 435 for Syria over the first 5 years of war (2011–2016) or 81 per 100,000 in CAR in 2010, a period far less violent than more recent years [15, 16]. Likewise, while mortality data is scarce, Mali has experienced the highest rate of United Nations’ peacekeepers killed, as well as the highest rate of aid workers killed in recent years [17].

There are several reasons for this discordance. The WHO has five categories of violent deaths: interpersonal violence, war, suicide, legal executions, and collective violence [18]. In the categories of homicide or interpersonal violence, Venezuela does have the highest rates among these five countries within the WHO database [19]. It is not clear why hundreds of killings of protesters and others in Venezuela by the government are not reflected in WHO’s violence data, which only shows interpersonal violence [20]. Thus, some discordance is related to definitions of violence specific mortality, and the reporting of such by individual governments.

In August 2018, a Google search using the phrases “Murder rates by country” and “Homicide rates by country” produced identical results for the top 9 results, with the first discrepancy at source 10. Clearly, these two terms are treated as synonyms in the Google search algorithm. A search on the phrases “Violent death rates by country” and “Violence specific mortality rate by country” returned the same top two results as the “Homicide” and “Murder” searches. Among the top 10 results, one other was the same and two more referenced WHO’s interpersonal violence data. Thus, 5 of the top 10 sources were functionally the same when using the phrase “violent mortality” or “violence specific mortality” versus “homicide” or “murder”, suggesting that the Google algorithm does not distinguish war deaths from interpersonal violence deaths in the way WHO does.

However, it is probable that larger issues of connectivity of individual societies with each other and with the outside world is playing a profound role in the deceptive results found in this exercise. Reviews regarding outbreak detection across the globe suggest this lack of connectivity affects detection of health problems in profound and tangible ways [21]. It is possible this lack of connectivity also affects the directing of humanitarian assistance. For example, in 2000 during the Kosovo crisis, the relief community spent 18 times more per affected beneficiary in Southeastern Europe than in Somalia, and at least 1000 times more per death when compared to Eastern DRC [22–24]. Being close to Europe, access to international communication, and being of political and military interest to major donors are factors likely associated with both donor motivation to spend, and media motivation to cover a crisis. The influence of these factors existing is likely greater in vicinity of wealthiest nations, such as with crises in Kosovo in 2000 and Syria in 2018.

Of note, the graduate students who undertook this task generally had work experience, had impressive academic credentials, are internet savvy, and are about to become humanitarian workers and reference librarians. Thus, their being misled by the internet on this issue suggests that most internet users would be as well. Aid workers, librarians and scholars need the skills to: find and understand the implications of definitions used by sources, understand synonyms used by search mechanisms, identify primary data sources, assess the independence of sources for triangulating findings, and to assess bias associated with social connectivity related information like that which flows with phone and social media access.

It appears the interacting of three factors (different definitions, internet search algorithms, and a society’s connectivity) make internet search results undervalue primary data sources, and in settings like CAR and Mali, primary mortality data sources appear to be lacking.

## Conclusion

There is a need to have standardized measures using standardized definitions in the world’s most acute crises. Such data is consistently collected for nutrition while often lacking for mortality [25]. In the absence of such data, the internet will likely continue to steer attention and compassion away from the world’s poorest and worst crises.
